# Molluscum contagiosum of the corneal limbus in an AIDS patient: a clinicopathological case report

**DOI:** 10.1186/s12886-022-02312-2

**Published:** 2022-02-21

**Authors:** Yukihiro Fujita, Satoru Kase, Susumu Ishida

**Affiliations:** Department of Ophthalmology, Faculty of Medicine Graduate School of Medicine, Hokkaido University, 7-chome, Kita 15 West, Kita-ku, Sapporo, 060-8638 Japan

**Keywords:** Molluscum contagiosum, Histopathology, Optical coherence tomography, AIDS

## Abstract

**Background:**

Molluscum contagiosum, a pox virus infection, is likely to occur in the eyelid skin; however, corneal involvements by molluscum lesions are extremely rare. We report a case of molluscum contagiosum arising in the corneal limbus in an untreated AIDS patient, together with anterior segment optical coherence tomography (OCT) findings and histopathology of the excised tumor.

**Case presentation:**

A 46-year-old man with AIDS was referred to our department for the management of an ocular lesion. Blood tests revealed an extremely low CD4+ T-cell count of 11 cells/μL, being strongly positive for anti-HIV antibody (591.36 S/CO) with a high copy number of HIV RNA (8070.0 × 100 copy/mL). Slit-lamp examination of his right eye showed a white nodule at the lower limbus. Anterior segment OCT findings of the nodule revealed a highly reflective elevated lesion, which was considered to involve the Bowman layer. The nodular lesion was excised from the limbus including the superficial corneal stroma, and then processed for histologic examination. Histopathology of the excised lesion showed acanthotic corneal epithelium containing swollen cells with eosinophilic inclusions known as molluscum bodies. He was diagnosed with molluscum contagiosum.

**Conclusions:**

Anterior segment OCT findings provide useful information for morphological evaluations of and preoperative strategies against molluscum contagiosum.

## Background

Molluscum contagiosum (MC) is well-known as a human skin infection, which is caused by a virus of the pox group. MC can spread by direct contact with infected persons or virus-contaminated objects. Molluscum lesions begin as dome-shaped, shiny bumps with a central dimple or whitish “core.” MC is likely to occur anywhere on the skin, common sites of which are the neck, armpits, chest, thighs, buttocks, genitals, and face, excluding the palms and soles. The number of bumps ranges from 1 to dozens, and they are often grouped together [1]. Ophthalmic involvements usually manifest as single or multiple lesions on the skin of the eyelids [2]; however, MC arising from keratoconjunctival sites is extremely rare. Indeed, only a few case reports of MC on the corneal limbus have been documented, with clinical data solely provided by slit-lamp examination [3, 4]. Therefore, clinical information on keratoconjunctival MC remains to be clarified.

Anterior segment optical coherence tomography (AS-OCT) is a non-invasive tool for morphological assessment of ocular tumors involving anterior segments such as the cornea, conjunctiva, and iris with high resolution and high reproducibility. We recently reported morphological assessments of a metastatic iris tumor by AS-OCT, which revealed useful clinicopathological correlations before and after treatments [5]. We herein report a case of MC arising in the corneal limbus, in which AS-OCT was employed for clinical diagnosis and to formulate a preoperative strategy.

## Case presentation

**Fig. 1:**
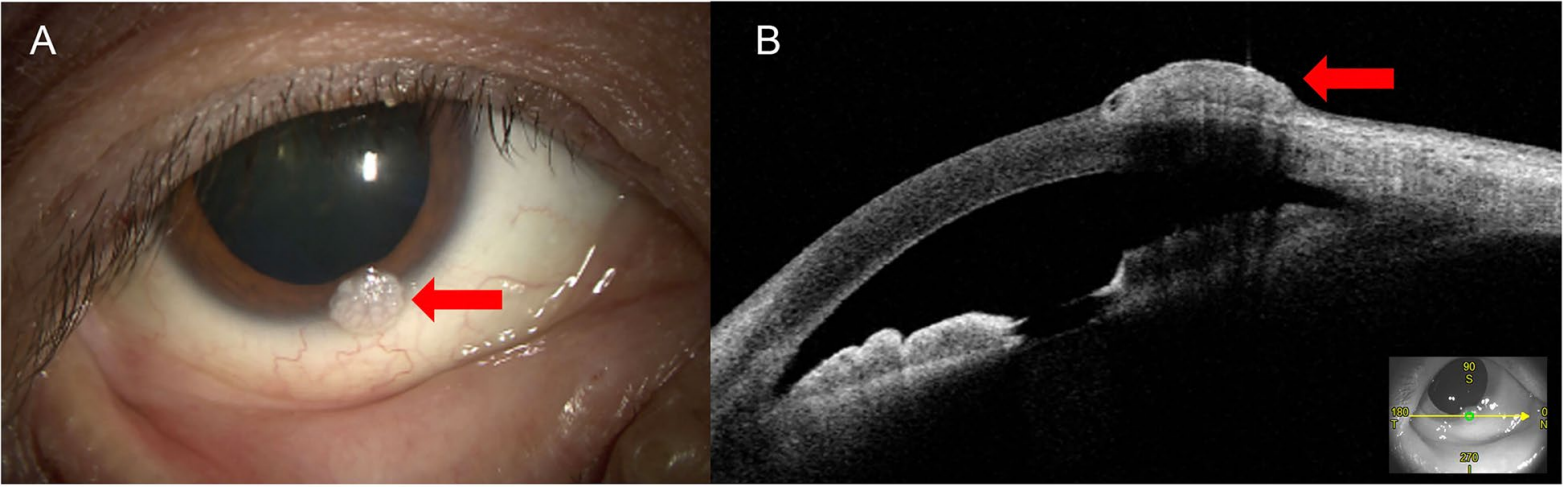
Clinical findings in a patient with molluscum contagiosum. **A** Slit-lamp examination of his right eye shows a white nodule of the limbus inferiorly. **B** A horizontal section of anterior segment optical coherence tomography depicts a highly reflective elevated tumor on the corneal limbus. A lower right insert image shows the direction of the cross section by infrared light

A 46-year-old man had noted a painless elevated lesion on his right eye for 8 months. He had been diagnosed with acquired immunodeficiency syndrome (AIDS) incidentally on examination for the comorbidity of *Candida* esophagitis 6 months ago, and had struggled with AIDS-related non-Hodgkin lymphoma. His CD4+ T-cell count was 11 cells/μL and strongly positive for anti-HIV antibody (591.36 S/CO) with a high copy of HIV RNA (8070.0 × 100 copy/mL) in the peripheral blood. There was no past medical or family history. Best corrected visual acuity was 20/20 with normal intraocular pressure in both eyes OU. A whitish nodule with an irregular surface, measuring 2 × 2 mm, was present inferiorly in the corneal limbus of his right eye (Fig. 1A, arrow). Slit-lamp examination showed no inflammatory signs such as hyperemia on the ocular surface. The intermediate optic media and fundus were unremarkable. AS-OCT (CASIA2; Tomey Corporation, ver.4A.1) showed a highly reflective elevated lesion with the horizontal section, which was considered to involve the corneal stroma (Fig. 1B, arrow). The tumor was completely excised together with incision of the superficial corneal stroma.

**Fig. 2:**
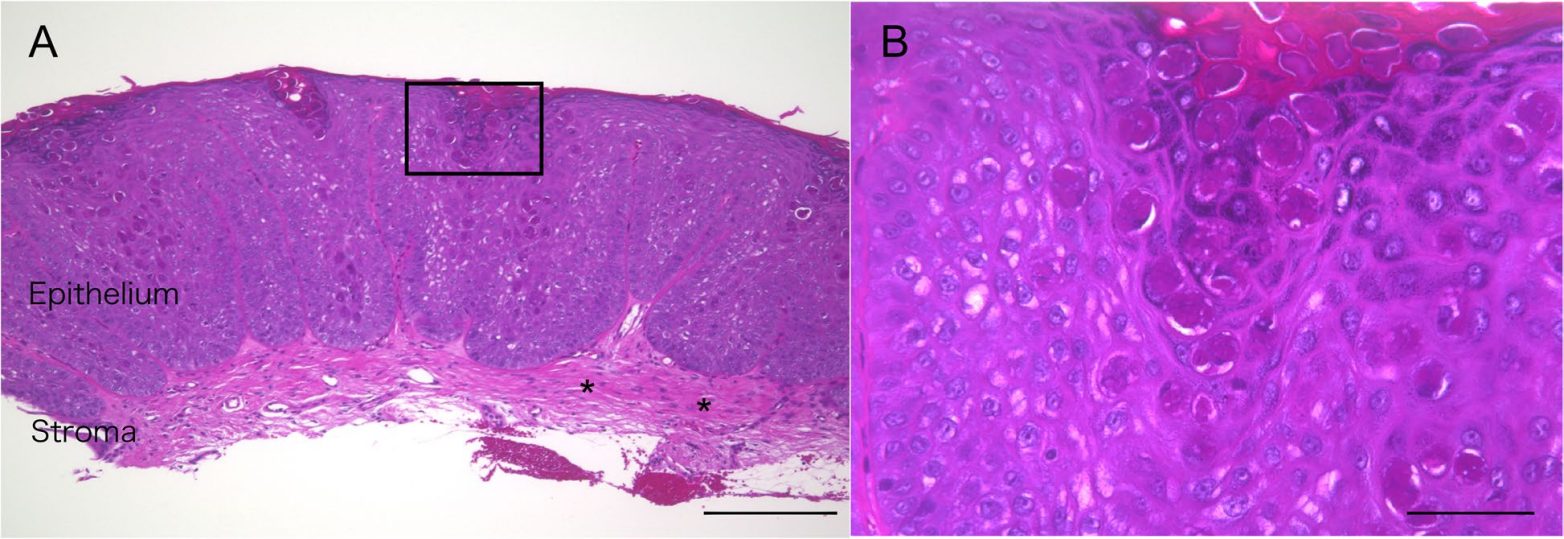
Histopathology of the excised tumor. **A** Photomicrograph, which is thought to be the cut section depicted by optical coherence tomography, shows acanthotic corneal epithelium together with superficial corneal stroma (asterisks), which is corresponding to the cut section of anterior segment optical coherence tomography. The objective lens is × 10. A bar indicates 100 μm. **B** High magnification, corresponding to the square in (**A**), shows swollen cells with inclusion bodies, known as molluscum bodies within the granular and horny layers, without inflammation. The objective lens is × 10. A bar indicates 50 μm

Pathologic examination showed a dome-shaped half split tumor, measuring 2 × 2 × 1 mm. Pathologic specimens were observed, and the pathological pictures were taken with the built-in camera under a Biorevo microscope (type: BZ-9000; Keyence, Tokyo, Japan). The pathological pictures were initially captured as JPEG image with 96 pixels, where the brightness was appropriately modified, without using any software. Each photograph was then incorporated into Photoshop CC^R^ (Adobe KK, Tokyo, Japan), and the marge image was created. Microscopic findings revealed complete resection of the acanthotic corneal epithelium (Fig. 2A) adjacent to the corneal stroma (Fig. 2A: asterisks). There were various swollen epithelial cells containing eosinophilic inclusions (molluscum bodies) within the granular and horny layers (Fig. 2B). In contrast, there were no inflammatory reactions including lymphocytic infiltration in the tissue. He was histologically diagnosed with MC arising from the corneal limbus. Two months later, the wound had healed with a smooth ocular surface.

## Discussion and conclusion

This MC case was an untreated AIDS patient with a low CD4 + T-cell count, indicating that he was severely immunocompromised. Among 43 HIV patients in Ethiopia, the overall prevalence of ocular manifestations was 25.3%, and 9 (2.6%) patients were complicated by MC [6]. Three out of the 9 patients were immunocompromised hosts with low CD4+ T cells less than 200 cells/μL, while MC was more common in AIDS patients with a CD4+ T-cell count over 200 cells/μL in peripheral blood [6]. However, the ophthalmic sites of MC were not clearly documented in the report. Basically, MC is more likely to arise on the eyelids [2] that are rich in blood vessels than on the cornea, which might be unaffected by the level of host immunity. Some reported MC arising on the corneal limbus [3, 4] like in the present case; however, the CD4+ T-cell count was not described. In this case, MC occurred on the corneal limbus, when the immune system was severely weakened. In addition, histopathology proved no infiltration of inflammatory cells or immune response in the corneal tissue, suggesting that immunosuppression allowed MC to grow on the cornea. These findings might indicate differences in susceptibility to viral infection of the corneal limbus compared with the eyelid in severely immunosuppressive hosts.

According to case reports of MC arising on the ocular surface, the tumor is typically located on the corneal limbus inferiorly, like in this case. Although the reason is unknown, the lower part of the cornea is physiologically scratched during blinking by Bell phenomenon, which might allow the virus to attach the lower cornea.

Surgical removal is generally recommended for MC because it could be a less invasive procedure with a short operative time, and also contributes to good cosmetic results and subsequent pathologic diagnosis [7]. However, it is mandatory to consider how deep MC lesions are on preoperative evaluation in order to avoid incomplete resection. AS-OCT offers cut sections of various anterior segment tumors non-invasively; however, AS-OCT findings of MC on the ocular surface have not been reported. In this case, since AS-OCT indicated that the hyper-reflective superficial corneal lesions involved tissue beyond the Bowman layer, the tumor was resected beyond the Bowman layer. The histopathology of the excised tumor proved complete resection of the tumor including superficial corneal stromal tissues. The corneal wound remained clear without recurrence 2 months later. Therefore, this case highlighted that AS-OCT provided useful information to determine a preoperative strategy that avoids the risk of incomplete resection.

There are limitations in this study. First, postoperative OCT was not available due to the patient’s death; therefore, it was impossible to evaluate morphological improvements in the cross-section of the cornea after resection. Second, since AS-OCT in this study employed swept-source AS-OCT (CASIA2), it remains unknown how spectral-domain OCT (SD-OCT) depicts the corneal MC. However, a recent study has reported that there was no significant difference in evaluation of the corneal morphology with the use of SS-OCT and SD-OCT [8]. Whether the findings of tumor infiltration into the cornea differ from SD-OCT is a future issue.

In conclusion, AS-OCT findings provide useful information for morphological evaluations of and preoperative strategies against molluscum contagiosum.

## Data Availability

The datasets used and analyzed in the current study are available from the corresponding author on reasonable request.

## Abbreviations

MC: Molluscum contagiosum
AS-OCT: Anterior segment optical coherence tomography
CD: Cluster of differentiation
HIV: Human immunodeficiency virus
AIDS: Acquired immunodeficiency syndrome
